# Melittin-derived peptides exhibit variations in cytotoxicity and antioxidant, anti-inflammatory and allergenic activities

**DOI:** 10.1080/19768354.2022.2099971

**Published:** 2022-07-18

**Authors:** Haesoo Jung, Yong Soo Kim, Da-Min Jung, Kyeong-Seob Lee, Jung-Min Lee, Kee K. Kim

**Affiliations:** a Department of Biochemistry, Chungnam National University, Daejeon, Republic of Korea; b Dong Seo Medical Research Institute, Namyangju-si, Gyeonggi-do, Republic of Korea

**Keywords:** bee venom, detoxification, anti-inflammatory activity, allergenic activity, antioxidant activity

## Abstract

Melittin is a major component of bee venom; it is widely used in traditional medicine because of its therapeutic effects, such as anti-inflammatory effects. However, melittin has limited medical applications owing to its adverse effects, such as high cytotoxicity. In this study, we investigated the physiological activities of various hydrolyzed melittin-derived peptides to eliminate the cytotoxicity of melittin and enhance its efficacy. The 2,2'-azino-bis(3-ethylbenzothiazoline-6-sulfonic acid) (ABTS) radical scavenging assay confirmed that melittin-derived peptides showed antioxidant activity comparable to that of melittin. Moreover, unlike melittin, which showed high cytotoxicity in the 3-(4,5-dimethylthiazol-2-yl)−5-(3-carboxymethoxyphenyl)−2-(4-sulfophenyl)−2H-tetrazolium inner salt (MTS) assay, the melittin-derived peptides showed negligible cytotoxicity. Among the melittin-derived peptides, the peptide composed of sequence TTGLPALISWIKRKRQQ (P1) showed inhibitory effects on the mRNA expression of inflammatory cytokines and phosphorylation of IκBα, similar to the effects of melittin in RAW 264.7 cells. Degranulation of RBL-2H3 cells was analyzed using a β-hexosaminidase release assay to confirm the allergenic activity of melittin and P1, which showed remarkably reduced allergenicity of P1 compared to that of melittin. These results indicate that P1 maintained the anti-inflammatory effects of melittin while reducing its cytotoxicity and allergic reactions. In conclusion, the melittin-derived peptide P1 efficiently decreased the adverse effects while maintaining the beneficial effects of melittin, making it suitable for therapeutic applications.

## Introduction

The search for active derivatives of natural products with beneficial physiological effects has various advantages, such as safety for consumption by humans and diversity of functional roles (Atanasov et al. 2021). However, natural extracts, including active ingredients, vary in their composition and may have side effects, such as toxicity, when used in humans, and show limitations in standardization (Sahoo et al. 2010). Therefore, efforts to identify active ingredients with minimum side effects are being made, by the standardization of natural products and additional processes including detoxification so that they can be safely used by humans. These efforts will reduce the limitations of using extracts of natural products and ensure safety for humans.

Bee venom is secreted by bees to protect their colonies (Orsolic 2012; Maitip et al. 2021). It is a mixture of proteins including melittin and peptides that exhibit various pharmacological activities (Lin and Hsieh 2020; Guha et al. 2021; Maitip et al. 2021). Melittin, which is composed of 26 amino acids, is known to have antibacterial, anticancer, and anti-inflammatory effects and is being actively used in oriental medicine clinical trials. However, the amphiphilic property of the melittin peptide causes cell membrane lysis, resulting in serious side effects like anaphylaxis (Lee and Bae 2016; Cherniack and Govorushko 2018). These side effects, due to melittin, limit the clinical application of bee venom. In addition to reducing the side effects of bee venom caused by melittin, the removal of phospholipase A2 and bioactive amines is necessary to maximize clinical application of bee venom, as they induce immune response (Wehbe et al. 2019; Carpena et al. 2020).

Inflammatory reaction is a normal response to tissue damage, pathogen infection, and chemical stimuli, and is initiated by the migration of immune cells to the site of damaged tissues and release of mediators (Chen et al. 2018). When macrophages are stimulated by pro-inflammatory substances such as lipopolysaccharide (LPS), TLR4 receptors recognize them and secrete cytokines through inflammatory signaling pathways, such as NF-κB signaling (Lu et al. 2008; Dorrington and Fraser 2019; Ciesielska et al. 2021). The reactive oxygen species (ROS), such as O^2-^, H_2_O_2_, OH^-^, and NO, react with biomolecules to induce molecular damage and oxidative stress, which causes inflammatory diseases (Aruoma 1998; Brieger et al. 2012; Checa and Aran 2020; Forman and Zhang 2021). Therefore, inhibition of intracellular ROS is necessary for anti-inflammatory activity, and the discovery of natural products with antioxidant and anti-inflammatory activity is important for treating and preventing various immune diseases.

In our previous study, we reported that detoxification by hydrolysis minimized cytotoxicity and allergic reactions due to bee venom, while maintaining its antioxidant and anti-inflammatory activities (Lee et al. 2021). The detoxification of bee venom was confirmed by hydrolysis of melittin protein, a major component of bee venom, into four different melittin-derived peptides. Among the four peptides identified during the detoxification process of bee venom, it was not confirmed which melittin-derived peptides had antioxidant and anti-inflammatory activities. In this study, we investigated the antioxidant, cytotoxic, anti-inflammatory, and allergenic activities of melittin-derived peptides.

## Materials and methods

### Cell culture and peptides

**Figure 1.**
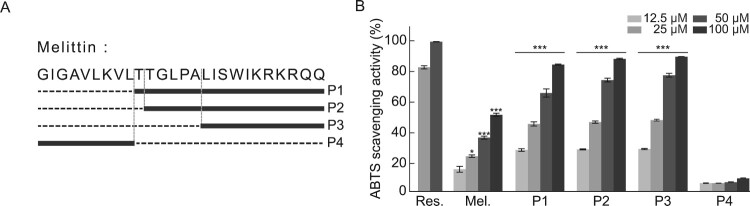
*In vitro* antioxidant activities of melittin and melittin-derived peptides. **(A)** Location of peptides in melittin protein. **(B)** ABTS radical scavenging activity of resveratrol (Res.), melittin, and melittin-derived peptides (P1–P4). Results are expressed as means ± SD (n = 3). *p < 0.05, ***p < 0.001.

Human pulmonary bronchial epithelial BEAS-2B, human epithelial cervix carcinoma HeLa, murine macrophage RAW 264.7, and rat basophilic leukemia mast RBL-2H3 cells were purchased from American Type Culture Collection (ATCC, VA, USA). BEAS-2B cells were maintained in bronchial epithelial basal medium (BEBM; Lonza, Basel, Switzerland). HeLa, RAW 264.7, and RBL-2H3 cells were maintained in DMEM supplemented with 10% heat-inactivated fetal bovine serum (Welgene, Gyeongsangbuk-do, Korea) and 1% penicillin and streptomycin (Welgene) at 37 °C in a humidified atmosphere of 5% CO_2_. Melittin and its derived peptides (Figure 1A) were synthesized by Peptron, Inc. (Daejeon, Korea).

### Free radical scavenging assay

The antioxidant activities of melittin and melittin-derived peptides were measured using the 2,2'-azino-bis(3-ethylbenzothiazoline-6-sulfonic acid) (ABTS) radical scavenging assay. ABTS^+^ radical solution was prepared by mixing equal volumes of 7 mM ABTS solution with 2.4 mM persulfate solution and allowing them to react in the dark at 25 °C for 24 h. Before this reaction, the ABTS^+^ radical solution was diluted with distilled water to obtain an absorbance of 0.7 ± 0.01 units at 650 nm. Melittin and melittin-derived peptides (20 µL each) at various concentrations were then reacted with 80 µL of ABTS^+^ radical solution for 4 min in the dark, and absorbance was measured at 650 nm using an EMax Plus microplate reader (Molecular Devices, San Jose, CA, USA). ABTS^+^ radical scavenging activity was calculated using the following formula: ABTS+ radical scavenging activity (%) = [1 − (Abs_sample_ − Abs_blank_ of sample) / Abs_control_] × 100, where Abs_control_ is the absorbance of ABTS^+^ radical solution diluted in water, Abs_sample_ is the absorbance of ABTS^+^ radical solution mixed with melittin or melittin-derived peptides, and Abs_blank_ of sample is the absorbance of water mixed with melittin or melittin-derived peptides. All experiments were performed in triplicates (n = 3).

### Cell viability assay

The effect of melittin and melittin-derived peptides on cell viability was evaluated using the CellTiter 96^R^ Aqueous One Solution Cell Proliferation Assay (Promega, Madison, WI, USA), according to the manufacturer’s instructions (Cambronero-Urena et al. 2021). BEAS-2B and HeLa cells were seeded (3,000 cells/well) in 96-well plates and incubated for 24 h. Culture medium containing melittin or melittin-derived peptides was added to the plates, and they were incubated for 24 h. Thereafter, 20 μL of MTS reagent was added, and absorbance was measured at 490 nm using a microplate reader (Molecular Devices EMax Plus). The formula used to calculate the cell viability is as follows:

Cell viability (%)={(A Sample−A [Blank]) / (A [Control])−A [Blank])}×100,

Where *A* is the absorbance for a given wavelength.

### RNA preparation and quantitative reverse transcription-PCR

RAW 264.7 cells (1 × 10^5^ cells/well) were seeded in 6-well plates and incubated for 24 h (Ahmed et al. 2020). Next, culture medium with melittin or melittin-derived peptides was added to the plates, and they were incubated for 24 h. Thereafter, lipopolysaccharide (LPS) was added to the medium at a final concentration of 1 μg/mL, followed by incubation for 6 h. The medium was completely removed, the cells were washed with cold PBS and lysed with 1 mL of RiboEx (GeneAll, Korea), and RNA was extracted using a Hybrid-R RNA purification kit (GeneAll). To synthesize cDNA, RNA was quantified using a Nabi UV/Vis nanospectrophotometer (MicroDigital, Korea), and 1 μL of random hexamer (100 pmol/μL) and 1 μL of dNTP mix (10 mM) were added to each RNA sample (1 μg). The total volume was adjusted to 10 μL using DEPC-treated water. The mixture was subjected to heating at 65 °C for 5 min and immediately cooled on ice. Thereafter, 1 μL M-MLV reverse transcriptase (Promega), 4 μL 5X M-MLV RT reaction buffer (Promega), 1 μL RNase inhibitor (Enzynomics, Korea), and 4 μL DEPC-treated water were added to each RNA sample, and the mixture was incubated at 25 °C for 10 min and then at 50 °C for 1 h.

The mRNA expression levels of inflammatory cytokines were analyzed using qRT-PCR. The reaction mixture contained 10 μL of 2X Prime Q-master Mix (GENET BIO, Korea), 1.5 μL of 10 pmol/μL forward primer, 1.5 μL of 10 pmol/μL reverse primer, 5 μL of DEPC-treated water, and 2 μL of cDNA (1/10 dilution); qRT-PCR was performed using AriaMx (Agilent, USA) as per the following conditions: 40 cycles of denaturation at 95 °C for 20 s, annealing at 58 °C for 20 s, and elongation at 72 °C for 20 s. The primers used for the qRT-PCR are listed in Supplementary 1.

### Immunoblot analysis

RAW 264.7 cells (1 × 10^5^ cells/well) were seeded in a 6-well plate and incubated for 24 h. Medium containing melittin or melittin-derived peptides was added, and the plates were incubated for 24 h. Thereafter, LPS was added to the medium at a final concentration of 1 μg/mL, followed by incubation for 1 h. To prepare protein samples, the medium was completely removed by aspiration, and the cells were washed with cold PBS and then lysed with lysis buffer [10 mM Tris-HCl (pH 7.5), 100 mM NaCl, 1 mM EDTA, 1 mM EGTA, 1% Triton X-100, 10% glycerol, and 0.1% SDS] with a protease inhibitor cocktail (Roche, Switzerland). The protein samples were sonicated and centrifuged at 16,000 × *g* for 10 min. The supernatant was then transferred to a new tube and used for immunoblot analysis. The protein samples were separated using sodium dodecyl sulfate-polyacrylamide gel electrophoresis (SDS-PAGE), and the separated proteins were transferred onto a nitrocellulose membrane (0.45 μm, Merck Millipore, USA). PBS-T with 5% skimmed milk was used as a blocking buffer for 1 h, and the nitrocellulose membrane was treated with anti-phospho-IκBα (9246S, 1:500, Cell Signaling Technology), anti-IκBα (L35A5, 1:500, Cell Signaling Technology), and anti-GAPDH (H86504M, 1:2,000, Meridian Life Science) antibodies diluted in blocking buffer for 12 h at 4 °C. The unbound antibodies were washed with PBS-T, and the antibodies bound to the nitrocellulose membrane were detected using the SuperSignal system (SuperSignal; Thermo Fisher Scientific), using horseradish peroxidase-conjugated secondary antibodies (Abcam, Cambridge, UK).

### β-Hexosaminidase release assay

The β-hexosaminidase release assay was performed as previously described. RBL-2H3 cells (5 × 10^4^ cells/well) were seeded in 96-well plates and incubated for 24 h. The medium was removed, and the cells were washed twice with siraganian buffer (119 mM NaCl, 5 mM KCl, 5.6 mM glucose, 0.4 mM MgCl_2_, 25 mM PIPES, 40 mM NaOH, 1 mM CaCl_2_, and 0.1% BSA, pH 7.2), and then incubated in 100 μL siraganian buffer supplemented with compound 48/80 (Sigma-Aldrich, St. Louis, MO, USA), melittin, or melittin-derived peptides for 1 h. Compound 48/80 was used as a positive control. The reaction was terminated by incubation at 4 °C for 10 min. The fluorescence of β-hexosaminidase in the supernatant was measured with a β-hexosaminidase activity assay kit (Cell Biolabs, USA) using a 2104 EnVision multilabel plate reader (PerkinElmer, USA) at emission (340 nm wavelength) and excitation (450 nm wavelength). The relative fluorescence units of β-hexosaminidase release were calculated by subtracting the fluorescence intensity of the control group from that of the experimental group.

### Statistical analysis

All the experiments had at least three independent biological replicates. The data are presented as mean ± standard error, and statistical analysis was performed using Student's *t*-test for significance.

## Results

### Antioxidant activity of melittin and melittin-derived peptides

Our previous study showed that the detoxification process can minimize the cytotoxicity and allergic reaction-inducing properties of bee venom while maintaining its anti-inflammatory functions. In addition, the amino acid sequences of four major peptides derived from melittin, a major component of bee venom produced through detoxification, were identified (Figure 1A) and their anti-inflammatory activities were investigated. ROS generated in biological processes react with biomolecules and cause various diseases, such as pathological aging (Valko et al. 2007; Scialo et al. 2017). The free radical scavenging ability of melittin and melittin-derived peptides, which are the main components of bee venom that act against these free radicals, was measured using the ABTS assay. To assess the antioxidant activity of melittin and its four derivatives, a comparative analysis of antioxidant activity was performed using resveratrol, which has an excellent antioxidant efficacy, as a positive control group. As shown in Figure 1A, with the exception of P4, which consists of nine amino acids of the N-terminal of melittin, other peptides, including melittin, had antioxidant efficacy comparable to that of resveratrol (Figure 1B). P1, P2, and P3, composed of the C-terminal peptide of melittin, showed similar antioxidant activity, and this trend was predictable because the peptide constituting P3 is commonly included in P1 and P2, suggesting that the antioxidant effect of melittin originates from the peptides constituting the C-terminal.

### Cytotoxicity of melittin and melittin-derived peptides

**Figure 2.**
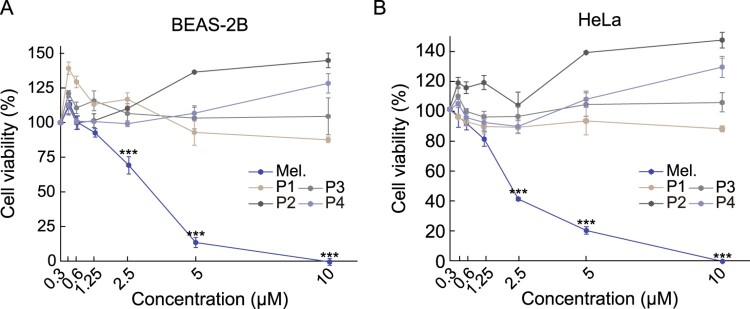
Comparison of cytotoxicity between melittin and melittin-derived peptides. **(A-B)** Cells were treated with specified concentrations of melittin and melittin-derived peptides for 24 h. Cell viability was determined using the MTS assay. Results are expressed as mean ± SD (n = 3). ***p < 0.001.

The clinical application of bee venom is limited owing to the high cytotoxicity of melittin. We analyzed the cytotoxicity of melittin-derived peptides using the MTS assay. As expected, melittin showed strong cytotoxicity in BEAS-2B cells at a concentration of 5 μM or higher (Figure 2A). Melittin also showed cytotoxicity in BEAS-2B and HeLa cells, with cellular activity of 75% and 60%, respectively, at a concentration of 2.5 μΜ (Figure 2A-B). In contrast, no cytotoxicity was observed in BEAS-2B and HeLa cells by melittin-derived peptides even at a concentration of 10 μM. These results suggested that melittin-derived peptides have significantly lower cytotoxicity than melittin.

### Anti-inflammatory activity of melittin and melittin-derived peptide P1

**Figure 3.**
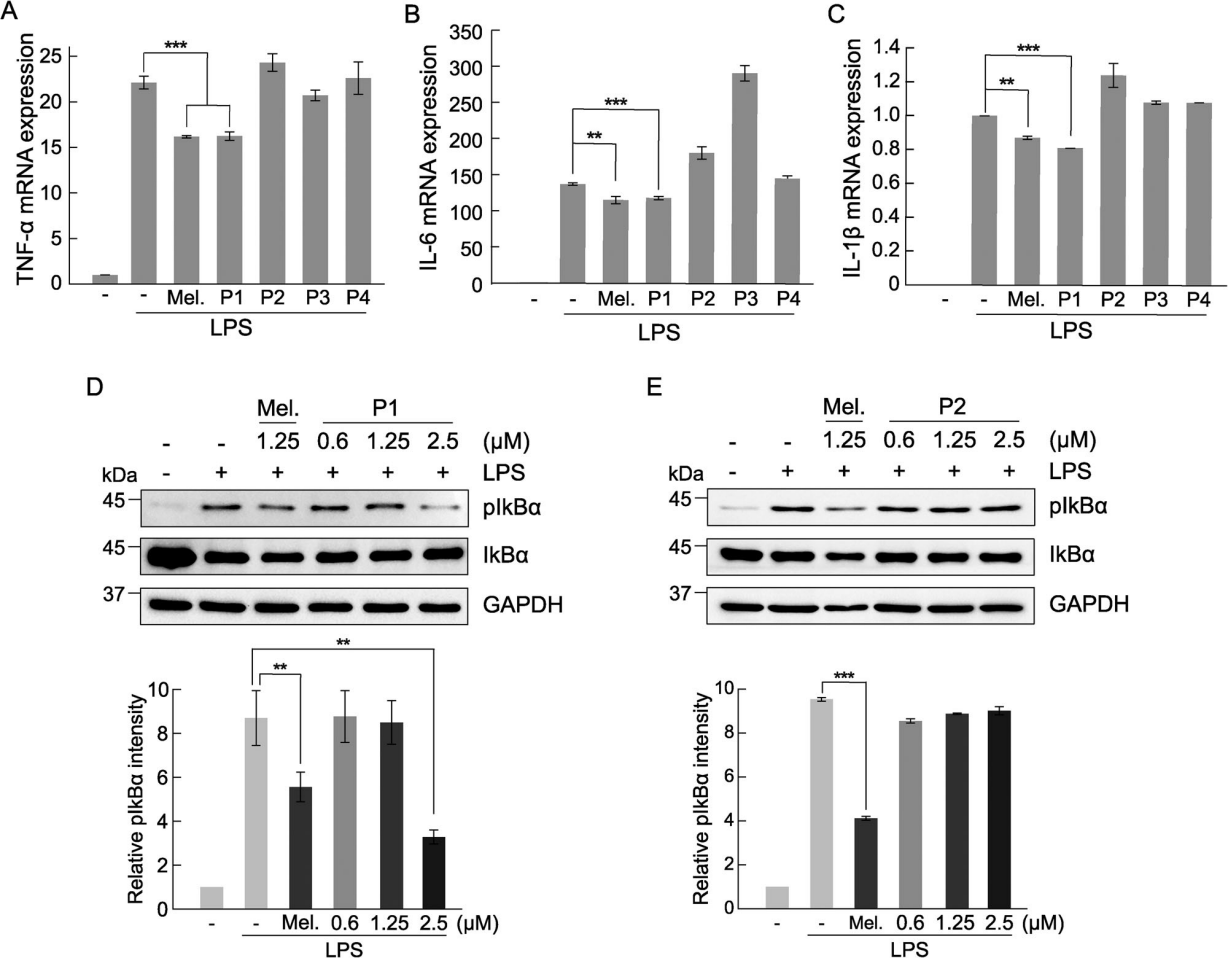
Comparison of anti-inflammatory activity between melittin and melittin-derived peptides. RAW 264.7 cells were pretreated with 1.25 μM melittin and melittin-derived peptides for 24 h and then incubated with LPS (1 μg/mL) for 6 h. qRT-PCR was performed to analyze the mRNA expression of the pro-inflammatory cytokines TNF-α **(A)**, IL-6 **(B)**, and IL-1β **(C)** Results are expressed as means ± SD (n = 3). **(D-E)** RAW 264.7 cells were pretreated with 1.25 μM melittin and indicated concentrations of peptide P1 and P2 for 24 h and then stimulated with LPS (1 μg/mL) for 1 h. The cell extracts were subjected to immunoblot analysis with anti-IκBα and anti-phospho-IκBα antibodies. Bar graphs show the relative expression of phospho-IκBα normalized with GAPDH. Results are expressed as means ± SD (n = 3). ***p < 0.001, **p < 0.01.

We investigated the anti-inflammatory effects of melittin and melittin-derived peptides. The expression of inflammatory cytokines, such as TNF-α, IL-6, and IL-1β, is increased through the inflammatory pathway inside the cell after the recognition of the specific TLR4 receptor of macrophages by an inflammatory signal, such as LPS (Standiford 2000; Liu et al. 2017). The expression of TNF-α, IL-6, and IL-1β in response to inflammation was increased by treatment with LPS in RAW 264.7 cells, and to confirm the anti-inflammatory efficacy of melittin and its hydrolyzed peptides, the mRNA expression levels of inflammatory cytokines were measured. The expression of all inflammatory cytokines was increased by LPS treatment, and significant inhibition of the expression of inflammatory cytokines by melittin and melittin-derived peptide P1 was observed, but not by other peptides (Figure 3A–C). Melittin-derived peptide P1 showed a concentration-dependent anti-inflammatory response at a concentration of 1.25 μM or higher, whereas in case of melittin, cytotoxicity was observed at a concentration of 1.25 μM or higher, making it difficult to confirm its anti-inflammatory activity (Figure 2A-B). Furthermore, the anti-inflammatory activity of P1 was confirmed by increase in the level of IκBα phosphorylation when the inflammatory reaction was induced by LPS in RAW 264.7 cells. The increased IκBα phosphorylation by LPS treatment was decreased slightly after treatment with melittin, and significantly by treatment with P1 at a concentration of 2.5 μM (Figure 3D). While IKbα phosphorylation induced by LPS treatment was effectively inhibited by P1, P2 in which one N-terminal threonine amino acid was removed from the P1 peptide did not show such inhibitory effect (Figure 3E). These results suggest that the inhibition of anti-inflammatory response of P1 is dependent on the inhibition of NF-κB signaling.

### Allergenic activity of melittin and melittin-derived peptides

**Figure 4.**
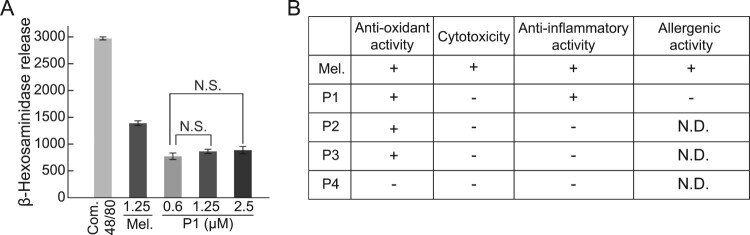
Comparison between degranulation activity of melittin and melittin-derived P1. **(A)** RBL-2H3 cells were treated with 1 mg/mL compound 48/80, 1.25 μM melittin, or indicated concentrations of P1 for 1 h. Compound 48/80 was used as a positive control for degranulation. Release of β-hexosaminidase was measured using fluorometric analysis. Results are expressed as mean ± SD (n = 3). N.S., no statistical significance. **(B)** Summary of activities of melittin and melittin-derived peptides. +, active. -, inactive. N.D., not determined.

Next, we examined the allergic response to melittin-derived peptide P1. The release of β-hexosaminidase was examined after the treatment of RBL-2H3 cells with melittin and P1. Compound 48/80, an oligomeric mixture of the condensation products of N-methyl-p-methoxyphenethylamine and formaldehyde, was used as a positive control to confirm the completion of degranulation reaction. The release of β-hexosaminidase did not increase with increasing P1 concentration (Figure 4A).

## Discussion

Bee venom is a natural product that is widely used medically because of its excellent pharmacological effects, such as anti-inflammatory activity. Various side effects of bee venom limit its application despite its excellent pharmacological properties (Zhang et al. 2018). Melittin has major pharmacological effects such as anti-inflammatory and anticancer effects. However, because of its amphipathic structure, it acts as a detergent that dissolves cell membranes and causes side effects such as anaphylaxis, edema, and fever (Son et al. 2007; Lee and Bae 2016). In this study, we discovered a melittin-derived peptide with reduced cytotoxicity, with consistent pharmacological activity, and investigated its efficacy (Figure 4B).

The discovery of effective substances with high antioxidant activity is important for the prevention of aging and diseases caused by intracellular oxidative stress (Tan et al. 2018). Our analysis of melittin, which is composed of 26 amino acids, and its derivatives revealed that P4, which is composed of nine N-terminal amino acids, did not show antioxidant activity. However, P3, composed of C-terminal 11 amino acids, P1, and P2 showed higher antioxidant activity than melittin. Considering that amino acids have antioxidant activities, such as glutamine (Liang et al.), arginine (R), tryptophan (W), and serine (S) (Tapiero et al. 2002; Amelio et al. 2014; Liang et al. 2018; Wu 2021), their presence in the C-terminal region of melittin could be responsible for the antioxidant properties of P1, P2, and P3.

Cytotoxicity due to the amphipathic structure of melittin causes quantitative limitations in its clinical application (Hong et al. 2019). Melittin-derived peptides with deletion of the N-terminus and C-terminus were non-cytotoxic due to the non-amphipathic structure, suggesting that quantitative limitations due to the cytotoxicity of melittin can be removed. Melittin-derived peptide P1, from which nine N-terminal amino acids of melittin were removed, showed anti-inflammatory activity similar to that of melittin. Interestingly, P2, which is P1 peptide with deleted N-terminal threonine (T), did not exhibit anti-inflammatory activity. In this study, we did not directly test the stability of each peptide. However, it is unlikely that the differences between P1 and P2 in cytotoxicity and anti-inflammatory are caused by different stability, because both P1 and P2 showed similar antioxidant activity. Additional studies on modification of the peptide to increase its stability and overall anti-inflammatory properties are required.

## Conclusion

We investigated and confirmed the physiological functions of melittin-derived peptides detected in detoxified bee venom, such as antioxidant and anti-inflammatory effects, and allergenic activity. P1, which is composed of the 17 C-terminal amino acids of melittin, did not show cytotoxicity and allergic reactions, but showed excellent antioxidant and anti-inflammatory activity comparable to that of melittin. These results demonstrated that melittin derivatives can overcome the limitations of bee venom for clinical application. Therefore, melittin-derived peptides could be valuable pharmacological substances for the prevention and treatment of inflammation and age-related diseases.

## Data Availability

The data that support the findings of this study are available from the corresponding author upon reasonable request.
